# The role of meditation in coping with stress during the COVID-19 lockdown: a cross-sectional study of undergraduates in India

**DOI:** 10.3389/fpsyt.2025.1573407

**Published:** 2025-09-24

**Authors:** Puja Munjal, Neetika Khurana, Manish Saggar, Lalit Kumar

**Affiliations:** ^1^ Department of Information Technology, Jagannath International Management School, Delhi, India; ^2^ Institute for Meditation & Inner Harmony (IMIH), Lisle, IL, United States; ^3^ Department of Psychiatry & Behavioral Science, Stanford University, Stanford, CA, United States; ^4^ Department of Physics, Hindu College, University of Delhi, Delhi, India

**Keywords:** COVID-19 pandemic, mental health, meditation, undergraduates, lockdown, stress, PSS-10, SOS meditation

## Abstract

**Background:**

The COVID-19 pandemic significantly impacted mental health worldwide, particularly among college students. This study aimed to evaluate the effects of regular meditation practice on stress and well-being during the COVID-19 lockdown in college students from Delhi, India.

**Methods:**

Data were collected from April 22 to June 3, 2020. Participants included college students aged 18–26, divided into two groups: those with regular meditation practice and those without prior meditation experience. Stress levels were measured using the Perceived Stress Scale (PSS-10). Statistical analyses included group comparisons and regression models to examine the relationship between meditation frequency, duration, and stress levels.

**Results:**

Compared to the meditation-naive group, participants in the meditation group reported significantly fewer adverse effects of the lockdown on their mental and emotional well-being. Stress levels were lower in the meditation group, and both the frequency and duration of meditation sessions were inversely associated with stress.

**Conclusions:**

Regular meditation practice appears to mitigate the negative impact of lockdown on mental health, reducing stress and promoting emotional well-being. These findings underscore the potential of meditation as an accessible, effective tool for stress management, particularly in challenging contexts such as the COVID-19 pandemic.

## 1 Introduction

The COVID-19 pandemic caused unprecedented disruptions globally, with college students particularly affected by sudden changes to their academic, social, and personal lives. In India, as occurred in many other countries, the government-imposed lockdown aimed at controlling the pandemic had a profound effect on the mental health of undergraduates, intensifying existing stressors and introducing new challenges related to academic uncertainty, isolation, and health concerns (1).

Previous research has extensively documented the psychological toll of the pandemic on students. For example, during the COVID-19 pandemic, research shows that medical and engineering students in India experienced heightened stress and anxiety due to the uncertainty surrounding exams and academic progress (2). Studies across various academic disciplines have similarly shown increased levels of anxiety, depression, and stress among undergraduates, who often turned to diverse coping mechanisms to manage their psychological distress (3–5). Sleep quality, another critical aspect of mental well-being, was also negatively affected during the lockdown, with nearly half of medical students reporting poor sleep quality during this period (6).

In addition to these general findings, research has shown that individual resilience and adaptive coping strategies played a pivotal role in mitigating the mental health impacts of the pandemic. Some students employed emotion-focused or avoidant coping behaviors, such as self-blame, venting, or distraction, to manage stress (5), while others relied on more constructive approaches, including meditation and exercise, to maintain their well-being (4, 7). Meditation, in particular, has gained attention as a promising tool for enhancing resilience and alleviating psychological distress during times of crisis (8–11).

Beyond India, global research has also highlighted the positive effects of meditation on coping with pandemic-induced stress. Several studies have shown that mindfulness, meditation, and other contemplative practices can effectively reduce stress and anxiety among students during the pandemic. For example, a survey of university students found that a brief online mindfulness and compassion-based intervention significantly decreased stress and anxiety while increasing self-compassion (10). Similarly, a randomized controlled trial with university students demonstrated that mindfulness-based interventions improved mental health outcomes among university students, including reduced stress and anxiety and enhanced well-being (11). Additionally, Yoga and meditation were also found to improve health-related quality of life in several domains, such as coping with stress and maintaining productivity during the pandemic (12). Moreover, a meta-analysis suggested that regular meditation could be an essential tool for boosting immunity and alleviating stress among students (1).

The present study aims to build on the existing literature by examining the effects of regular meditation on the mental health of undergraduate students during the COVID-19 lockdown. Specifically, we compare two groups of Indian undergraduates: one group that engaged in daily meditation practice and another that was meditation-naive. Our objective is to explore the impact of COVID-19 and its associated lockdowns on the mental health of these students and assess whether regular meditation provided relief from stress and anxiety, serving as a valuable coping mechanism during a period of heightened uncertainty.

In this study, we also focus on a specific form of focused-attention meditation, known as SOS meditation, which remains understudied in the scientific literature despite its widespread practice in South Asian traditions. SOS meditation is a form of focused attention meditation, where practitioners are taught to sit in silence, withdraw attention from bodily sensations and external stimuli, and focus inward on a sustained sense of inner awareness, often accompanied by the silent repetition of a calming or meaningful word (13).

SOS meditation can be situated within the phenomenological matrix proposed by Lutz et al. (14), which organizes contemplative practices based on dimensions such as object orientation, dereification, and meta-awareness (14). SOS meditation is characterized by a high degree of object orientation (toward internal awareness), high dereification (intentional detachment from bodily and sensory identification), and a narrow attentional aperture. The practice aligns most closely with the Focused Attention (FA) family of meditations, though it is distinctive in its active inward attention and use of a repetitive internal verbal anchor (13).

While previous research has demonstrated that meditation can improve mental well-being, few studies have examined whether the frequency and duration of meditation sessions modulate stress reduction. This approach allows us to examine not only whether meditation works, but whether more frequent and sustained practice yields greater benefits during a period of global crisis. Specifically, this study aims to test three specific hypotheses:

- H1: Participants in the meditation group would be less impacted by the COVID-19 lockdown than those in the meditation-naive group.
- H2: Participants in the meditation group would report lower stress levels compared to the meditation-naive group, as measured by self-reports on the PSS-10.
- H3: The higher the frequency/duration of meditation sessions, the lower the perceived stress.

By examining these hypotheses, this study provides new insights into meditation’s role as a coping strategy during the COVID-19 pandemic, highlighting the significance of meditation frequency/duration in promoting stress resilience.

## 2 Methods

### 2.1 Participants

The study involved 180 participants aged between 18 and 26. Of the 180 participants initially enrolled, only 148 were enrolled in undergraduate studies. Thus, we limited further analysis to these 148 participants.

Out of 148, 86 undergraduate students were in the non-meditator group enrolled from the Hindu college in Delhi. The other 62 undergraduate students formed the meditator group. The meditator group was mainly recruited from the cities near Delhi (aka National Capital Region or NCR). All participants in the meditator group follow the SOS meditation practice.

Participants in the meditation group were recruited through the Delhi meditation center for SOS meditation and affiliated online groups during the lockdown period. As part of the screening survey, participants were asked: (1) “Do you meditate regularly?” (2) “Which type of meditation do you practice?” and (3) “Do you practice any other meditation or relaxation techniques?” Only participants who answered that they practiced SOS meditation exclusively were included.

During the data collection period (April–June 2020), India was under a nationwide lockdown, and all academic institutions had shifted to online instruction. Therefore, all participants—regardless of prior residential status (hosteller or day scholar)—were residing at home with their families at the time of the survey.

All data collection was conducted through secure online surveys. Informed consent was obtained digitally before survey initiation.

### 2.2 Meditation practice

SOS meditation is a focused attention meditation technique that promotes calmness and concentration (13). Seated comfortably, participants are instructed to close their eyes gently and focus their attention approximately 8–10 inches before them while staying mentally alert. To maintain focus, participants silently repeat a self-chosen calming word or phrase steadily, preventing their minds from wandering. This practice sometimes led to visual experiences, such as flashes or circles of light, interpreted as signs of heightened focus. Participants were encouraged to sustain their attention on the center of these experiences, enhancing the calming and peaceful effects of the meditation.

### 2.3 Measurements

#### 2.3.1 Perceived stress scale

Stress levels were assessed using the PSS-10, a widely used self-report questionnaire that measures perceived stress over the past month. Participants rated their stress on a 10-item scale, with higher scores indicating greater levels of perceived stress (15).

#### 2.3.2 COVID-19 lockdown impact

Participants completed a self-reported, single-item questionnaire to assess the impact of the COVID-19 lockdown on their mental state. The question, “Is your current mental state and emotional well-being the same as before the lockdown for COVID-19 (Yes/No)?” allowed for an evaluation of perceived changes in mental well-being due to the pandemic.

#### 2.3.3 Demographic data

Information on participants’ demographic characteristics, including sex, age, self-employment status, and marital status, was collected to examine potential group differences and to control for these factors in analyses. Self-employment referred to participants who reported freelance, tutoring, online sales, or part-time entrepreneurial activities—common among urban undergraduates in the NCR region. As demographic variables (age, sex, employment) differed between groups, they were statistically controlled in all inferential analyses.

#### 2.3.4 Coping strategies

Participants were asked: “Which of the below activities best describes your approach for coping with stress and maintaining happiness during the lockdown period?” Options included meditation, exercise, video games, or other activities. Participants could also indicate a second most preferred activity using the same format. This item was used to identify both the primary and secondary coping strategies adopted during the lockdown (see **Supplementary Tables 1**, **2** for descriptive distributions for each group).

#### 2.3.5 Frequency of coping strategies

For their chosen primary activity, participants reported frequency of practice. Responses were recorded as number of days per week.

#### 2.3.6 Duration of coping strategies

Participants then reported the average daily duration of the selected coping activity. Responses were provided in minutes per day.

#### 2.3.7 Meditation experience (meditator group only)

Participants who identified meditation as their primary coping strategy also reported their meditation history using the item: “Since how many days are you practicing meditation?” Responses were recorded as total duration of prior practice (days).

### 2.4 Statistics

All statistical analyses were conducted using SPSS version 29.0.2.0. Independent samples t-tests and cross-tabulation analyses were conducted to examine group differences in demographic characteristics (age, sex, marital status, and self-employment status).

For our first hypothesis, which posited that the COVID-19 lockdown would have less impact on meditators compared to non-meditators, we used cross-tabulation analysis, followed by nominal regression to control for covariates (age, sex, marital status, and self-employment).

To test our second hypothesis regarding group differences in perceived stress, as measured by total PSS-10 scores, we conducted an Analysis of Variance (ANOVA) with covariates. This analysis allowed us to assess whether meditators reported significantly lower stress levels than non-meditators while controlling for potential demographic confounders.

For our third hypothesis, we examined whether meditation frequency/duration was negatively related to perceived stress using nonparametric bivariate correlation analysis (Spearman’s rho).

## 3 Results

### 3.1 Group differences in demographics

**Table 1** provides participant demographics. An independent samples t-test was conducted to compare age differences between the groups. A significant effect was observed (t(146)=-2.62, p= 0.01), such that participants in the meditator group (mean age (SD) = 21.29 (2.04) years) were significantly older than the non-meditator group (mean age (SD) = 20.59 (1.12) years).

**Table 1:** Participant demographics.

Group	Demographics	N	Description	Group differences
Non-meditators	Age	86	18–24 years	t(146)= -2.62, p = 0.01
	Sex		63 M, 23 F	χ² =5.4, p = 0.02
	Marital Status		1 Married	None
	Self Employment		6	χ² = 11.47, p < 0.001
Meditators	Age	62	18–26 years	
	Sex		34 M, 28 F	
	Marital Status		2 Married	
	Self Employment		17	

Cross-tabulation analyses were conducted to compare demographic characteristics (sex, marital, and self-employment status) between the meditators and non-meditators. For sex, a significant difference was observed between groups (χ²(1, N = 148) = 5.4, p = 0.02). Specifically, 28/62 of the meditator group were female compared to 23/86 of the non-meditator group. Similarly, group differences were observed for self-employment status (χ²(1, N = 148) = 11.47, p < 0.001), such that 17/62 of the meditator group were self-employed, whereas 6/86 of the non-meditator group. No group differences were observed for marital status.

Thus, age, sex, and self-employment were included as covariates in further analyses.

### 3.2 The COVID-19 lockdown less impacted meditators as compared to non-meditators

We conducted a cross-tabulation analysis to examine whether participants in the meditator group reported a significantly lesser negative impact on their mental state and emotional well-being due to the COVID-19 pandemic compared to the non-meditator group.

A significant association was found between the group (meditator vs. non-meditator) and the reported impact on mental and emotional well-being (Pearson χ²(1, N = 148) = 20.93, p < 0.001). Specifically, 77.4% of participants in the meditator group reported that their mental and emotional well-being remained unchanged or was minimally impacted, compared to 39.5% in the non-meditator group.

A nominal regression analysis was conducted to account for potential confounding variables, i.e., age, sex, and employment status. After adjusting for these covariates, the meditator group reported a significantly lesser negative impact on mental and emotional well-being (β = 1.563, p < 0.001), suggesting that the observed group differences were unrelated to demographic factors.

These findings support our hypothesis that regular meditation practice is associated with a reduced negative impact on mental and emotional well-being during the COVID-19 pandemic, independent of age, sex, and employment status.

### 3.3 Meditators experienced lower stress levels compared to non-meditators

We conducted a univariate general linear model analysis to test whether participants in the meditation group reported lower stress levels compared to the non-meditator group. The PSS-10 total score was used as the dependent variable, with group (meditator vs. non-meditator) as a random effect and age, sex, and employment status as covariates.

Descriptive statistics showed that the PSS-10 total scores for the meditation group ranged from 0 to 34, with a mean of 17.32 (SD = 6.455). For the non-meditator group, scores ranged from 4 to 38, with a mean of 20.10 (SD = 7.336). The skewness and kurtosis for PSS-10 scores were within acceptable bounds for normality (See **Table 2**).

**Table 2:** Summary table.

Variable	Group	Mean	SD	Median	Range	IQR	Skewness	Kurtosis
PSS-10 Score	Non-meditators	20.10	7.34	20.00	4-38	10	0.09	-0.36
	Meditators	17.32	6.46	16.50	0-34	7	0.40	0.99
Number of times coping activity was performed per week (Frequency)	Non-meditators	5.96	1.31	7.00	2-7	2	-1.11	0.65
	Meditators	5.25	1.92	5.50	1-7	3	-0.70	-0.72
Total time spent in coping activity per week (Duration; in minutes)	Non-meditators	282.74	119.39	315.00	30-420	225	-0.34	-1.05
	Meditators	164.25	109.93	150.00	15-420	135	0.91	0.34

**Figure 1:**
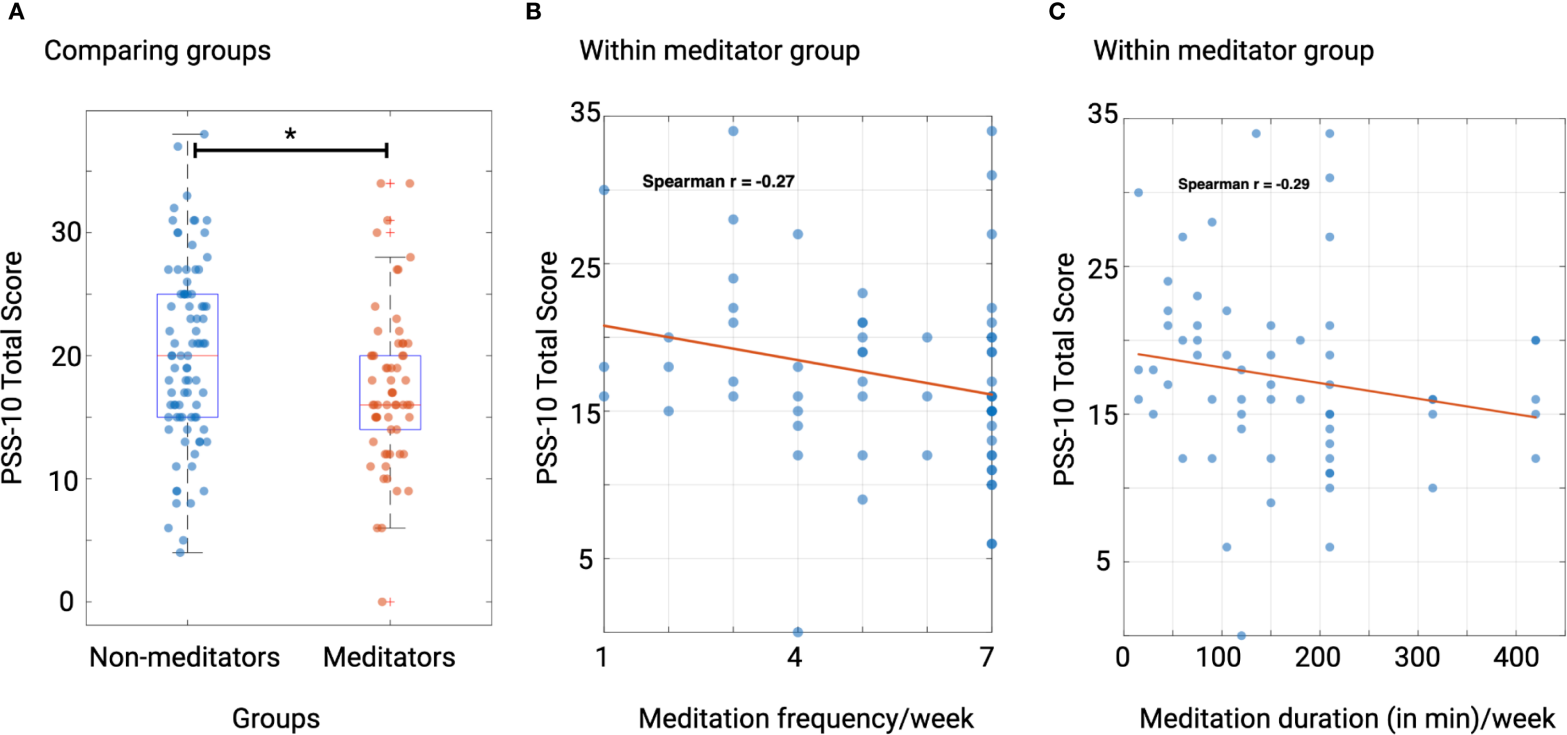
Regular meditation practice is associated with reduced perceived stress. **(A)** Group differences in PSS-10 total score, meditators reported less stress than non-meditators, after controlling for age, sex, and employment status (F(1,143) = 8.16, p = 0.005, Partial η² = 0.054). **(B, C)** Within the meditation group, more meditation (frequency and total duration/week) was associated with reduced stress levels. Specifically, the correlation between PSS-10 scores and frequency of meditation was r_s_ = -0.27, p = 0.03. Similarly, the correlation between PSS-10 scores and total time spent in meditation per week was r_s_ = -0.29, p = 0.02.

The univariate analysis revealed a significant effect of group on PSS-10 total scores, indicating that participants in the meditation group reported significantly lower stress levels than those in the non-meditator group after controlling for age, sex, and employment status (F(1,143) = 8.16, p = 0.005, Partial η² = 0.054). **Figure 1A** provides box plots for these results.

None of the covariates was a significant predictor of stress levels.

These results support our hypothesis, suggesting that regular meditation practice is associated with reduced perceived stress, independent of demographic factors.

### 3.4 Meditation frequency and duration are linked to less stress

To test our third hypothesis—that within the meditation group, those who meditated more would report lower stress levels—we examined the correlations between PSS-10 total scores and (1) frequency of meditation (number of times meditated per week) and (2) total time spent in meditation per week (in minutes). For this analysis, we employed Spearman’s rank correlation, a non-parametric approach suitable for variables with mild departures from normality (**Table 2**).

The analysis revealed significant negative correlations between PSS-10 total scores and both frequency and quantity of meditation. Specifically, the correlation between PSS-10 scores and frequency of meditation was r_s_ = -0.27, p = 0.03, with a 95% confidence interval (CI) of -0.5 to -0.01. Similarly, the correlation between PSS-10 scores and total time spent in meditation per week was r_s_ = -0.29, p = 0.02, with a 95% CI of -0.515 to -0.03. Scatter plots are shown in **Figures 1B, C**.

We also explored whether total lifetime meditation experience (months of prior practice) was associated with stress scores but found no significant correlation (Spearman’s ρ = -0.131, p = 0.318). This suggests that recent weekly meditation practice may be more strongly associated with stress reduction than prior experience, although larger samples are needed to test this conclusively.

Altogether, these findings support our hypothesis, suggesting that higher frequency and greater time spent meditating each week are associated with reduced perceived stress levels within the meditation group.

## 4 Discussion

The findings of this study highlight the beneficial effects of regular meditation on mitigating the psychological impact of the COVID-19 lockdown on undergraduate students in India. Consistent with previous research (e.g., 10, 11), students who engaged in regular meditation were less negatively impacted by the lockdown and reported significantly lower stress levels compared to non-meditators. These results suggest that meditation may serve as a valuable tool for building resilience and alleviating psychological distress during times of crisis.

Participants in the meditation group reported significantly less negative impact on mental and emotional well-being during the lockdown than non-meditators, even after controlling for age, sex, and employment status. This finding aligns with global studies demonstrating that mindfulness and meditation can effectively buffer against stress and enhance emotional stability in challenging circumstances (12). The present results extend this body of evidence to Indian undergraduates, highlighting that meditation—specially forms rooted in indigenous traditions—may serve as culturally congruent and accessible strategies for stress reduction and emotional resilience during crises.

Consistent with our hypothesis, meditation group participants reported significantly lower PSS-10 stress levels than non-meditators, independent of age, sex, and employment status, suggesting that regular meditation may foster stress resilience. The effect size, while modest (partial η² = 0.054), suggests that meditation could be an effective complementary intervention to help students manage stress. In a context where academic pressures and uncertainties surrounding exams and career prospects are pervasive (2, 6), meditation could offer a practical means of enhancing mental well-being among students.

Further, meditation frequency and duration were significantly associated with lower stress scores within the meditation group, supporting our hypothesis and highlighting a putative ‘dose-response’ effect. Specifically, higher frequency and total time spent meditating each week were linked to lower perceived stress, consistent with previous research emphasizing the importance of regular practice in achieving stress reduction (1). The results underscore the potential benefit of not only engaging in meditation but also doing so consistently and with adequate duration to maximize its positive effects.

In the meditators group, meditation was uniformly selected as the first-choice coping strategy, while music and exercise were the most common second-choice activities. Interestingly, these same strategies—music and exercise—were also the most frequent first and second choices in the non-meditators group. This overlap suggests that the observed differences in stress levels between groups are unlikely to be driven by these shared strategies. Instead, the findings underscore the potential unique role of SOS meditation in promoting stress resilience.

In this study, participants practiced a specific form of focused attention meditation, known as SOS meditation, which emphasizes awareness of the blank space in front of them (with eyes closed) rather than on bodily sensations. Unlike more popular mindfulness practices that focus on the breath—where participants become highly aware of sensory inputs, such as the tactile sensation of air passing through the nostrils—SOS meditation trains participants to direct their attention toward an external point of blankness, detached from immediate bodily sensations. This technique requires participants to continually refocus whenever distracted by thoughts, cultivating attention regulation and cognitive flexibility. By fostering detachment from sensory inputs and physical awareness, SOS meditation may help participants reduce stress by enhancing attentional control and regulation, potentially promoting resilience. Future research could explore these unique aspects by comparing SOS meditation with other practices, such as mindfulness or loving-kindness meditation, to examine differential effects on stress reduction and resilience.

### 4.1 Limitations and future directions

This study has several limitations. First, while our sample size was sufficient to detect moderate effects, it may not capture smaller or interaction-level effects. Caution is advised when generalizing these findings, and future studies with larger and more diverse samples are needed to replicate and extend these results. Second, the reliance on self-reported data may introduce biases related to subjective reporting. Future studies could validate these findings by incorporating objective stress measures, such as physiological biomarkers (e.g., heart rate variability, resting heart rate, etc.). Third, participants in the meditation and non-meditation groups were recruited from different colleges within Delhi and its surrounding National Capital Region (NCR). Although both groups were drawn from similar urban academic settings within the same metropolitan region, unmeasured institutional or micro-geographic differences may have influenced stress levels independently of meditation practice. Future studies using within-institution or matched sampling are warranted to rule out such effects more definitively. Fourth, although we collected information on coping strategies, their distribution did not allow for formal covariate analysis. However, since both groups reported similar non-meditation coping activities (particularly music and exercise), we believe the observed group differences in stress are meaningfully linked to the presence or absence of meditation practice. Fifth, our sample consisted solely of undergraduate students in India, potentially limiting the generalizability of the findings. Future research could replicate this study across diverse demographic and cultural contexts to assess the broader applicability of meditation as a coping strategy. Sixth, we observed group differences in age, sex, and employment status. To mitigate bias, we included these variables as covariates in all statistical models. However, this study was not designed as a matched case-control study. Future research should consider matched designs to better isolate the effects of meditation from demographic confounders. Seventh, although the current study used a quantitative design, future research would benefit from incorporating qualitative methods (e.g., interviews or open-ended surveys) to explore participants’ lived experiences with meditation and their perceived mechanisms of stress reduction. Such approaches would offer richer insight into how and why meditation works, particularly in a culturally specific context. Eighth, because this study was conducted using a cross-sectional design, we cannot infer causality between meditation and stress reduction. It is possible that lower-stress individuals are more likely to engage in meditation, or that both meditation practice and reduced stress are influenced by a third, unmeasured factor. Future studies should employ longitudinal and experimental designs to determine directionality. Ninth, we used a single-item measure to assess the perceived impact of the lockdown on emotional well-being, which may limit reliability and sensitivity. A more comprehensive, validated scale would provide a more nuanced understanding of this construct in future studies. Finally, while this study focused on meditation frequency and duration, exploring additional factors, such as meditation type and guidance quality, could provide further insights into optimizing meditation as a mental health intervention.

### 4.2 Conclusions

In sum, this study provides evidence that regular meditation practice, especially with greater frequency and duration, can reduce perceived stress and mitigate the mental health impacts of the pandemic-related lockdowns among undergraduates. These findings suggest that meditation could be a valuable, accessible tool for students facing heightened stressors and uncertainties. Institutions may consider incorporating meditation programs to support students’ mental well-being, fostering resilience in challenging times.

## Data Availability

The (anonymized) raw data supporting the conclusions of this article will be made available by the authors, without undue reservation.
