# Supplementary material for: The role of meditation in coping with stress during the COVID-19 lockdown: a cross-sectional study of undergraduates in India
Source: Front Psychiatry. 2025 Sep 24;16:1573407. doi: 10.3389/fpsyt.2025.1573407 (PMC12504295; doi:10.3389/fpsyt.2025.1573407)
Supplement: Supplementary file 1 [file SupplementaryFile1.docx]

### Supplementary Information

**Supplementary Table 1: First and second choice for coping activity for participants in the meditators group**

| First choice for coping activity |  |  |
| --- | --- | --- |
|  | N | % |
| Meditation | 62 | 100.00% |
|  |  |  |
|  |  |  |
| Second choice for coping activity |  |  |
|  | N | % |
| Exercise | 28 | 45.20% |
| Music | 21 | 33.90% |
| None | 4 | 6.50% |
| Not available | 2 | 3.20% |
| Spiritual Lectures | 2 | 3.20% |
| All | 1 | 1.60% |
| Cooking | 1 | 1.60% |
| Dancing | 1 | 1.60% |
| Family time | 1 | 1.60% |
| Studying | 1 | 1.60% |
|  |  |  |

**Supplementary Table 2: First and second choice for coping activity for participants in the non-meditators group**

| First choice for coping activity |  |  |
| --- | --- | --- |
|  | N | % |
| Exercise | 37 | 43.00% |
| Music | 37 | 43.00% |
| Movies | 5 | 5.80% |
| Family time | 3 | 3.50% |
| Reading | 2 | 2.30% |
| Spiritual Gratitude | 1 | 1.20% |
| Video Games | 1 | 1.20% |
|  |  |  |
|  |  |  |
| Second choice for coping activity |  |  |
|  | N | % |
| Music | 35 | 40.70% |
| Exercise | 29 | 33.70% |
| None | 9 | 10.50% |
| Movies | 4 | 4.70% |
| Family time | 2 | 2.30% |
| Friends | 2 | 2.30% |
| Video Games | 2 | 2.30% |
| Not available | 1 | 1.20% |
| Reading | 1 | 1.20% |
| Yoga | 1 | 1.20% |
|  |  |  |
